# Massive Hemoptysis in a Patient With Eisenmenger Syndrome, Polysplenia and Transverse Liver

**DOI:** 10.1002/ccr3.70313

**Published:** 2025-03-20

**Authors:** Mohsen Shafiepour, Behnam Dalfardi, Ali Nemati, Sina Bakhshaei, Elahenaz Parsi mood

**Affiliations:** ^1^ Clinical Research Development Unit Afzalipour Hospital, Kerman University of Medical Sciences Kerman Iran; ^2^ Advanced Thoracic Research Center, Tehran University of Medical Sciences Tehran Iran; ^3^ Assistant professor of Hematology and Oncology Afzalipour Hospital Research Center‚ Kerman University of Medical Sciences Kerman Iran; ^4^ Internal Medicine Resident UHS SoCal MEC Temecula California USA; ^5^ Cardiovascular Research Center, Institute of Basic and Clinical Physiology Sciences Kerman University of Medical Sciences Kerman Iran

**Keywords:** Eisenmenger syndrome, hemoptysis, polysplenia, pulmonary hypertension, transverse liver

## Abstract

Hemoptysis is defined as blood‐streaked sputum from the lower parts of the respiratory tract. Hemoptysis, even in small amounts, is a frightening and alarm sign for possible underlying conditions such as infections, pulmonary diseases, neoplastic conditions, cardiovascular alterations, vasculitis, traumatic events, hematological derangements, and iatrogenic or drug‐induced events. The initial step in the evaluation of hemoptysis is to determine the source of bleeding. Herein, we report an unusual case of massive hemoptysis in a young patient with polysplenia and Pulmonary Artery Hypertension (PAH) in the setting of Eisenmenger syndrome. Chest radiography was suggestive of multiple lung opacities bilaterally. Chest Computed Tomography (CT) revealed a non‐heterogeneous mass‐like lesion measuring 4 × 5.2 × 5.6 cm in the superior segment of the inferior lobe of the left lung, concerning for an accessory spleen. The patient underwent Video bronchoscopy, which showed tracheomalacia and active bleeding in the left main bronchus. The bleeding was controlled by the Argon Plasma Coagulation (APC) technique. Bronchoalveolar lavage (BAL) was negative for acid‐fast bacilli on staining and on culture. After stabilization, the patient was discharged home on medical management for PAH. On two‐week follow‐up, imaging revealed resolution of the pulmonary mass‐like lesion. Our report highlights the importance of bronchoscopy in determining the bleeding source in patients with hemoptysis and managing it via the APC technique.

**Summary**

- Eisenmenger syndrome is a severe form of cyanotic congenital heart disease characterized by large anatomical shunts, leading to a complex multisystem disorder.
- Hemoptysis has been documented as a significant manifestation of this syndrome. Additionally, Polysplenia syndrome (PSS) and transversus liver are rare congenital anomalies with an incidence of approximately 1 in 250,000 live births.
- Most affected individuals do not survive beyond the early neonatal period, as the conditions are frequently associated with severe cardiac and biliary abnormalities. The co‐occurrence of these anomalies with Eisenmenger syndrome is exceptionally rare and has not been reported in the medical literature to date.

## 1 Introduction

Hemoptysis is defined as blood‐streaked sputum from the lower parts of the respiratory tract. The initial step in the evaluation of hemoptysis is to determine the source of bleeding. Pseudohemoptysis, which refers to blood expectoration via a source other than the bronchial or pulmonary system, should be ruled out by history and physical examination [1].

Hemoptysis, even in small amounts, is a frightening and alarming sign. The clinical spectrum ranges from minor blood‐stained sputum to major bleeding causing respiratory failure and hemodynamic instability. Underlying causes may vary from benign self‐limiting conditions to severe and potentially life‐threatening conditions. In terms of severity, hemoptysis is considered scant when it presents with bleeding < 5 mL, mild when < 20 mL, and moderate when > 20 mL, while massive hemoptysis is defined as a bleeding amount of 100 mL/24 h or more [2].

Hemoptysis may derive from multiple underlying conditions, such as infections, pulmonary diseases, neoplastic conditions, cardiovascular alterations, vasculitis, traumatic events, hematological derangements, and iatrogenic or drug‐induced events [3].

In adults, acute respiratory tract infections (e.g., bronchitis, pneumonia), bronchiectasis, asthma, chronic obstructive pulmonary disease, and malignancy are the most common etiologies. Tuberculosis (TB) is a major cause of hemoptysis in endemic regions and in developing countries [4, 5]. However, in industrialized areas, bronchial carcinoma and bronchiectasis are more common culprits.

Pulmonary artery hypertension (PAH) is a serious condition causing progressive obstruction and obliteration of the pulmonary vascular bed. PAH is a rare cause of hemoptysis, which is responsible for 0.2%–4% of the cases. However, hemoptysis is a relatively more common finding in patients with Eisenmenger syndrome [1].

In the past, depending on the severity and etiology of hemoptysis, several management strategies were recommended, including supportive care, surgical resection, and lung transplant. Currently, the more commonly used strategy is bronchial artery embolization (BAE). In this technique, a particulate material is injected into angiographically identified abnormal bronchial arteries, helping in hemostasis. BAE is usually well tolerated; however, recurrent bleeding is commonly associated with the procedure [6].

According to the 2022 ESC guideline for PAH and recent articles, Prostacyclin analogs (epoprostenol, iloprost, treprostinil, beraprost, selexipag), PDE5 inhibitors (sildenafil and tadalafil), Riociguat, endothelin receptor antagonists (Ambrisentan, bosentan, and macitentan), and a novel treatment via bone morphogenetic protein receptor 2 (BMPR2), Nuclear Factor κβ, can partially treat PAH [7, 8].

Herein, we report a case of hemoptysis in a patient with Eisenmenger syndrome and polysplenia. We aim to portray the importance of bronchoscopy as both a diagnostic and therapeutic tool, which helped us identify the source of bleeding and manage it via the BAE technique.

## 2 Case Description

### 2.1 History

A 34‐year‐old female with a past medical history of Eisenmenger syndrome in the setting of a Ventricular Septal Defect (VSD), who presented with 10‐day history of cough, hemoptysis (about 300 mL/day), pleuritic chest pain, and exertional dyspnea. She did not report any fever, gum bleeding, or epistaxis. She was admitted to the hospital emergency department and initial management consisted of a focus on efficient stabilization, Advanced cardiac life support (ACLS) implemented as soon as possible. The non‐bleeding lung was protected by turning the patient to the bleeding side so the blood was isolated to the bleeding lung due to gravity, and the non‐bleeding side remained intact and fully aerated. Then oxygen therapy, keeping the patient at rest and suppressing cough, nebulized treatment was done. We also reserved FFP and packed cells for later evaluation. Then CXR and primary laboratory studies included type and cross‐matching of blood, complete blood counts and differential, coagulation profile, electrolytes, liver function tests, urinalysis, and arterial blood gas were sent.

### 2.2 Examination

On physical examination, the patient was tachypneic and tachycard., and hypoxic, with an oxygen saturation of 85% in room air. Chest auscultation revealed left upper lobe crackles and a holosystolic murmur in the lower sternal border. Also, she was found to have clubbing in her hands bilaterally.

## 3 Methods (Differential Diagnosis, Investigations)

**FIGURE 1:**
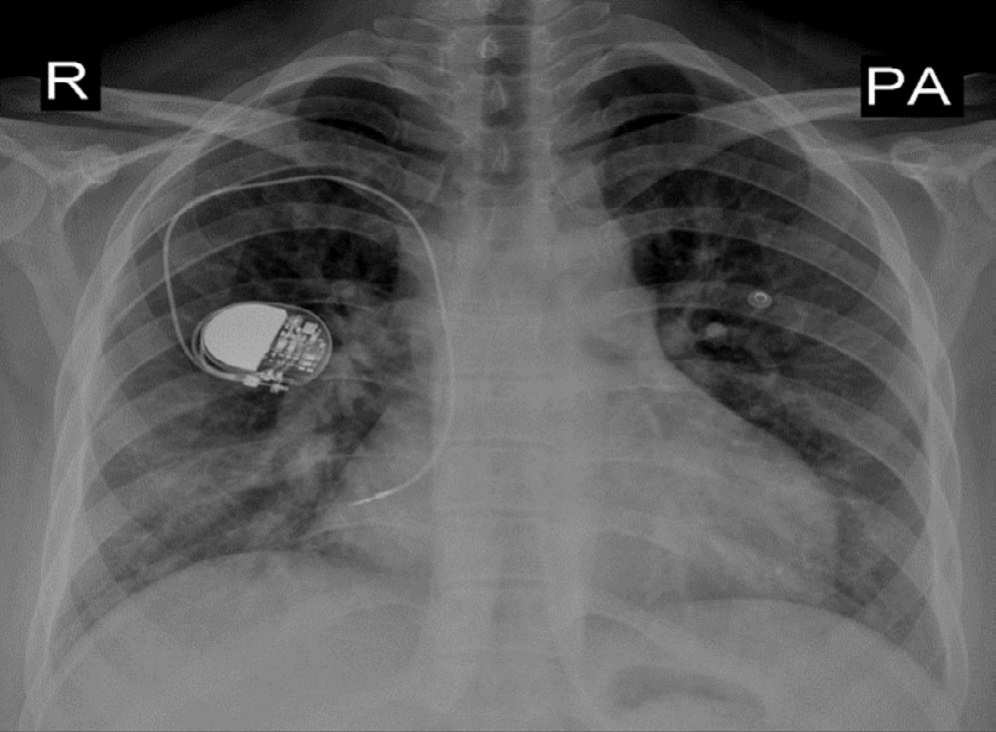
Chest X‐ray PA view revealed non homogenous opacity in both lungs. Cardiomegaly due to Right ventricle and both atrial enlargement. A single chamber pace maker in right hemi thorax with tip of lead in RA(His).

**FIGURE 2:**
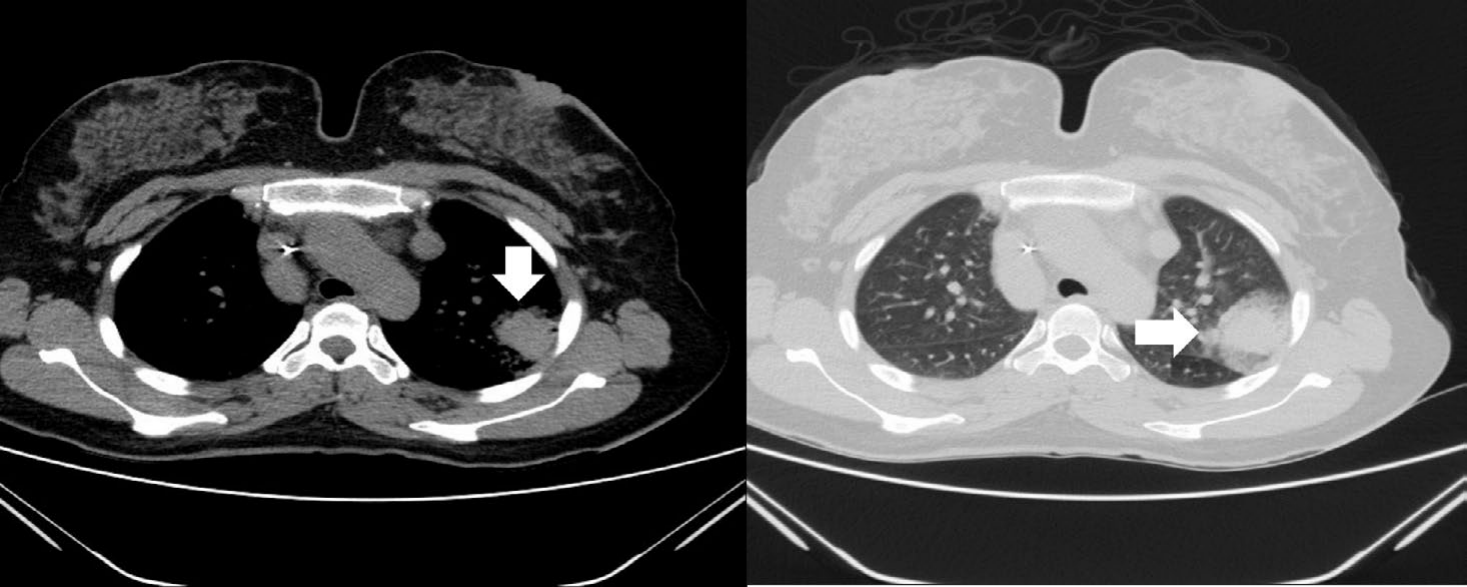
Chest CT showed non heterogeneous mass like lesion measuring 4 × 5.2 × 5.6 cm in superior segment of inferior lobe of left lung that in histologic report results show that inflammatory process due to old TB.

**FIGURE 3:**
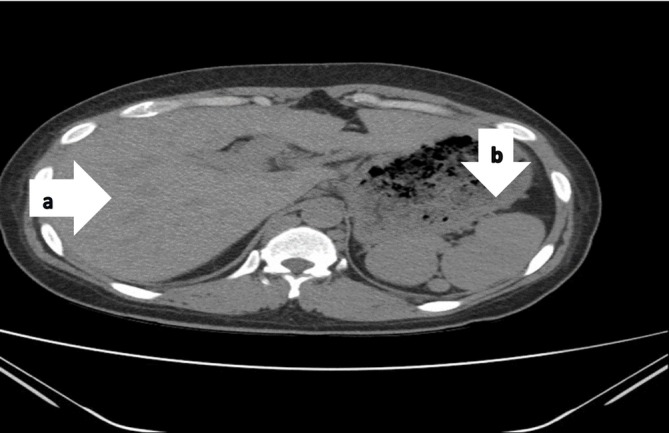
(a) Abdominal CT showing a large transverses liver in both upper quadrants of abdomen. (b) Poly splenia in LUQ, no tumor or metastases was seen in liver and spleens.

Laboratory findings were significant for microcytic anemia (Hemoglobin 10.6 g/dL), elevated erythrocyte sedimentation rate (ESR) at 23 mm/h, and negative sputum for both Acid Fast Bacilli (AFB) stain and geneXpert. Chest radiograph revealed non‐homogeneous opacities in both lungs (Figure 1). Computed tomography (CT) Chest showed a non‐heterogeneous mass‐like lesion measuring 4 × 5.2 × 5.6 cm in the superior segment of the inferior lobe of the left lung (Figure 2), in addition to a transversus liver and multiple spleens being noted (Figure 3). Echocardiography was remarkable for mild Pulmonary valve insufficiency, Pulmonary artery pressure (PAP) of 80 mmHg, abnormal septal motion, large membranous VSD, severe right ventricular (RV) enlargement, and left ventricular Ejection fraction (LVEF) of 55%.

**FIGURE 4:**
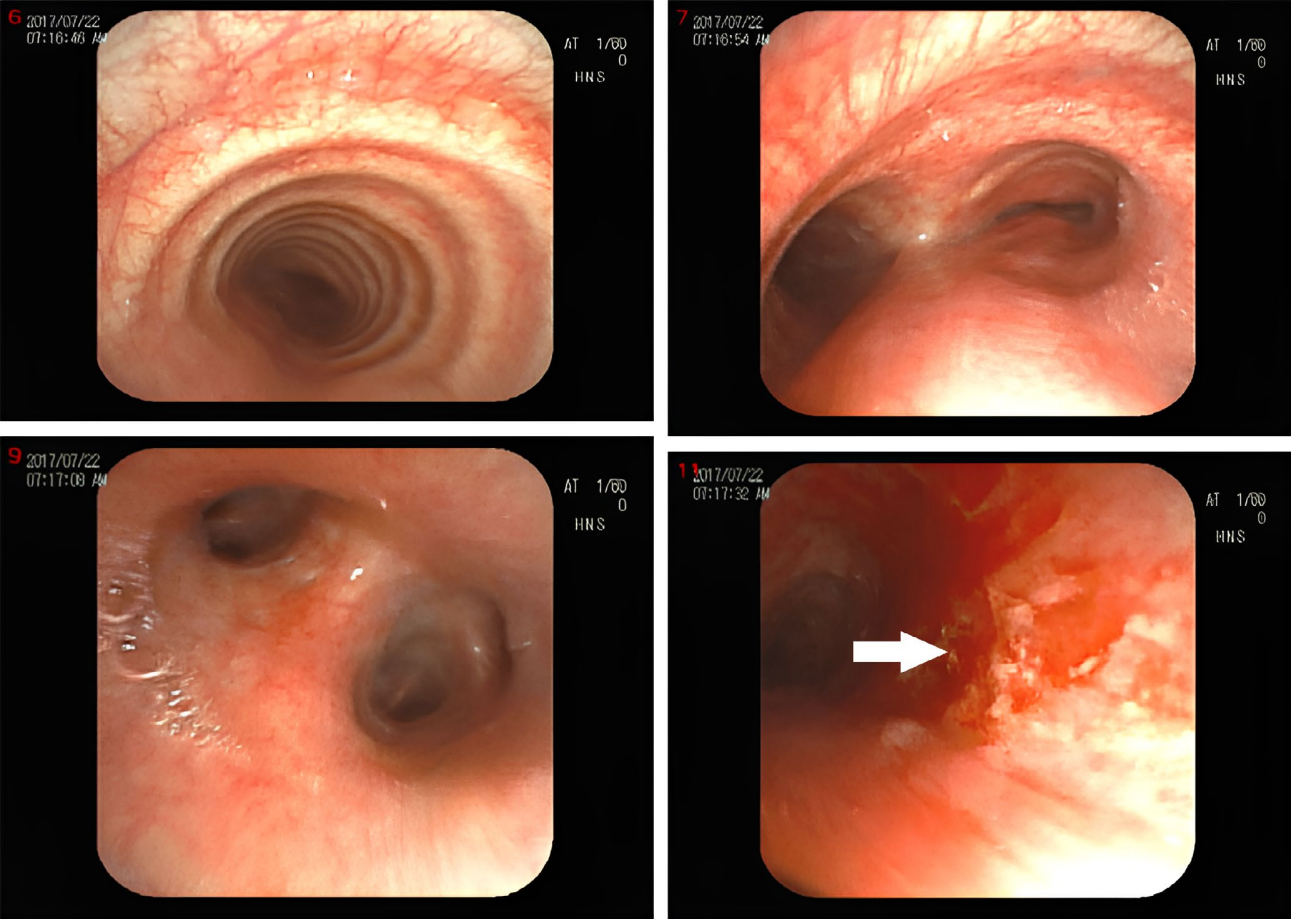
Bronchoscopic findings revealed hemorrhagic lesion in left upper segments of inferior lobe. In the images tracheomalasia and congestion is seen. In last image bleeding source in left main broncos is seen.

To identify the source of bleeding, a videoronchoscope was performed, which revealed tracheomalacia and active bleeding in the left main bronchus, which was controlled by APC (Figure 4). Bronchoalveolar lavage (BAL) was sent for cytology, anaerobic culture, Ziehl‐Neelsen stain, and GeneXpert to detect Mycobacterium TB, all of which were unremarkable.

The biopsy‐guided CT was taken from a nonheterogeneous mass in the superior segment of the inferior lobe of the left lung, and the pathologist reported the inflammatory process due to aspiration pneumonia (Figure 2).

## 4 Conclusion and Results (Treatment and Follow Up)

After stabilization, the patient was discharged on maintenance medications to control PAH, for example, diuretics, and was advised to follow up regularly with her pulmonologist. On a two‐week follow‐up, the patient remained asymptomatic. Lung examination was clear bilaterally, and repeat CXR was consistent with the resolution of the lung opacities.

## 5 Discussion

Hemoptysis is a serious complication of PAH that is rarely reported in end‐stage patients. The incidence of hemoptysis in PAH patients remains uncertain [6]. In 75% of the patients, chest CT scan shows evidence of bronchial artery hypertrophy. The number of dilated bronchial arteries correlates with the severity of PAH. The presence of hypertrophied bronchial arteries increases the risk of hemoptysis in PAH patients [9].

Diagnosis of pulmonary hypertension can be established by Right Heart Catheterization (RHC) and revealing elevated mean pulmonary arterial pressure (mean PAP) ≥ 25 mmHg at rest. Recommended pharmacologic treatment for PAH includes vasodilators, prostanoids, nitric oxide, phosphodiesterase inhibitors, endothelin receptor antagonists, and anti‐coagulants treatment [10]. Bronchial artery embolization is suggested as an immediate emergency procedure in patients with PAH and severe hemoptysis or as an elective intervention in patients with frequent episodes of mild‐to‐moderate hemoptysis. Anticoagulant therapy in patients with PAH and hemoptysis should be postponed. Interventional Bronchoscopy and APC are useful tools to control the bleeding. APC is an electrosurgical technique similar to laser or electrocautery, which is used during bronchoscopy procedures to ablate malignant airway tumors, control hemoptysis, remove granulation tissue from stents or anastomoses, and treat a variety of benign disorders [11]. However, when Bronchoscopy and APC fail to control massive hemoptysis, surgery solves the problem. Surgery remains the procedure of choice in the management of massive hemoptysis secondary to iatrogenic PA rupture, chest trauma, and aspergilloma resistant to other therapeutic options [12]. Lung resection can manage very massive hemoptysis, but the mortality rate is high [13].

Our case is the first report of hemoptysis in a patient with PAH and multiple spleens. Nugraha et al. in 2021 reported a 25‐year‐old woman with a past history of PAH who presented with hemoptysis (400 mL) and shortness of breath. Her examination revealed jugular venous dilatation, right ventricular heaving, accentuated 2nd heart sound, and a grade 3/6 pansystolic murmur best heard at the left lower sternal border. Further workups showed elevated PAP at 98 mmHg, a non‐reactive O_2_ test, observation of hemoptysis suspected to be associated with pulmonary hypertension, suspected Hospital Acquired Pneumonia (HAP), and hypokalemia. The treatments included sildenafil, digoxin, furosemide, iloprost nebulizer, Aspar K, ceftazidime, and ciprofloxacin [10].

Our patient had an atypical presentation of hemoptysis and pleuritic chest pain, and it was challenging to pinpoint the cause of bleeding, especially in the setting of concomitant polysplenia. Hemoptysis is usually an end‐stage presentation of PAH and has a poor prognosis. Given the severity of this condition, prompt and aggressive management is crucial to achieve the best outcome. The takeaway point from our report is to highlight the importance of bronchoscopy in determining the bleeding source in patients with hemoptysis and to manage the bleeding in a timely manner via the APC technique.

## Author Contributions

**Mohsen Shafiepour:** resources, supervision, writing – original draft. **Behnam Dalfardi:** resources, writing – original draft, writing – review and editing. **Ali Nemati:** resources, writing – original draft. **Sina Bakhshaei:** conceptualization, writing – review and editing. **Elahenaz Parsi mood:** conceptualization, writing – original draft.

## Ethics Statement

This is a case report and ethical approval was waived according to Medical ethics committee of Kerman university of medical sciences. Furthermore, the patient received all routine care and all identifying materials are removed to respect anonymity.

## Consent

We took informed written consent of the patient to report her report, additionally we ensured that patient's information would not be disclosed.

## Conflicts of Interest

The authors declare no conflicts of interest.

## Data Availability

Supporting data of this case are available from the corresponding author upon reasonable request.
